# Long-Term Retinal PEDF Overexpression Prevents Neovascularization in a Murine Adult Model of Retinopathy

**DOI:** 10.1371/journal.pone.0041511

**Published:** 2012-07-20

**Authors:** Virginia Haurigot, Pilar Villacampa, Albert Ribera, Assumpcio Bosch, David Ramos, Jesus Ruberte, Fatima Bosch

**Affiliations:** 1 Center of Animal Biotechnology and Gene Therapy, School of Veterinary Medicine, Universitat Autònoma de Barcelona, Bellaterra, Spain; 2 Departments of Biochemistry and Molecular Biology, School of Veterinary Medicine, Universitat Autònoma de Barcelona, Bellaterra, Spain; 3 CIBER of Diabetes and Associated Metabolic Disorders (CIBERDEM), Barcelona, Spain; 4 Department of Animal Health and Anatomy, School of Veterinary Medicine, Universitat Autònoma de Barcelona, Bellaterra, Spain; University of Sydney, Australia

## Abstract

Neovascularization associated with diabetic retinopathy (DR) and other ocular disorders is a leading cause of visual impairment and adult-onset blindness. Currently available treatments are merely palliative and offer temporary solutions. Here, we tested the efficacy of antiangiogenic gene transfer in an animal model that mimics the chronic progression of human DR. Adeno-associated viral (AAV) vectors of serotype 2 coding for antiangiogenic Pigment Epithelium Derived Factor (PEDF) were injected in the vitreous of a 1.5 month-old transgenic model of retinopathy that develops progressive neovascularization. A single intravitreal injection led to long-term production of PEDF and to a striking inhibition of intravitreal neovascularization, normalization of retinal capillary density, and prevention of retinal detachment. This was parallel to a reduction in the intraocular levels of Vascular Endothelial Growth Factor (VEGF). Normalization of VEGF was consistent with a downregulation of downstream effectors of angiogenesis, such as the activity of Matrix Metalloproteinases (MMP) 2 and 9 and the content of Connective Tissue Growth Factor (CTGF). These results demonstrate long-term efficacy of AAV-mediated PEDF overexpression in counteracting retinal neovascularization in a relevant animal model, and provides evidence towards the use of this strategy to treat angiogenesis in DR and other chronic proliferative retinal disorders.

## Introduction

Pathological retinal neovascularization is a feature of diabetic retinopathy and other ocular disorders characterized by retinal hypoxia [1]. The abnormal growth of blood vessels in the eye can threaten vision through several deleterious complications, such as intravitreal hemorrhage or retinal detachment. As in other tissues, retinal angiogenesis depends on the balance between pro- and anti-angiogenic factors. In retinal hypoxia there is a disruption of such balance due to intraocular increases of proangiogenic molecules, such as Vascular Endothelial Growth Factor (VEGF) [2]. Therefore, a therapeutic approach could be designed aimed at counteracting the disease-induced excess of angiogenesis stimulators by increasing the intraocular concentration of angiogenesis inhibitors. Intraocular gene transfer is an attractive strategy to achieve this, as long-term protein expression has repeatedly been reported in rodents [3], dogs and non-human primates [4] after a single gene transfer to the retina. Moreover, three independent clinical trials for an inherited form of blindness have recently confirmed the feasibility and safety of ocular gene transfer in humans [5]–[7].

Pigment epithelium-derived factor (PEDF) was originally isolated from a human retinal pigment ephitelium (RPE) cell culture [8] but has subsequently been reported to be expressed in several other tissues [9]. In the eye, PEDF expression is regulated by hypoxia in an opposite manner to VEGF [10]. Indeed, an inverse correlation between vitreous PEDF and VEGF levels in patients with diabetic retinopathy has been described [11], [12]. Amongst its many reported actions, PEDF shows powerful antiangiogenic properties, with specificity for newly formed vessels [13]. PEDF has also been proven to be neuroprotective in animal models of neuronal degeneration [14]–[16]. In addition, PEDF has been reported to have anti-inflammatory properties [17].

To test the efficacy of PEDF in counteracting the retinal neovascularization characteristic of diabetic retinopathy, we established a gene therapy protocol based on the transfer to the mouse retina of the human PEDF gene using adeno-associated viral vectors of serotype 2 (AAV2), which efficiently transduce the retina [18]. As a model of diabetes-like retinopathy and retinal neovascularization we used transgenic mice overexpressing IGF-I in the retina (TgIGF-I) [19], [20]. Due to retinal IGF-I accumulation, these mice show increased levels of intraocular VEGF and vascular alterations that progress from a non-proliferative retinopathy to neovascularization that causes retinal detachment [19], [20]. This model offers advantages compared to other commonly used models of retinal angiogenesis, such as the ischemia-induced retinopathy model, also known as retinopathy of prematurity model (ROP), in which vessels regress at approximately 3 weeks of age [21]. Although the ROP model has proven successful for proof-of-concept studies, it has not allowed the evaluation of the long-term efficacy of antiangiogenic gene transfer in counteracting chronic neovascularization. In contrast, in the IGF-I transgenic retinas, neovascularization occurs and worsens as animals age [19], [20]. This is a key aspect when evaluating therapies for a chronic disease such as diabetic retinopathy, in which years may go by between the onset of vascular alterations and the appearance of pathological neovascularization.

Here we show that AAV2-mediated gene transfer leads to high and sustained (up to 18 months) PEDF overexpression after a unique intravitreal delivery of the vector to TgIGF-I mice. PEDF treatment resulted in a reduction in intravitreal neovascularization, capillary density, and incidence of retinal detachment. PEDF also mediated the down regulation of VEGF in TgIGF-I-treated retinas, contributing to the decrease of downstream effectors of retinal neovascularization. Altogether, these results demonstrate the long-term efficacy of AAV2-mediated PEDF gene transfer in an animal model in which the development of retinal neovascularization follows a disease progression that mimics the human pathology.

## Results

### AAV2 mediated expression of PEDF in TgIGF-I retinas

**Figure 1:**
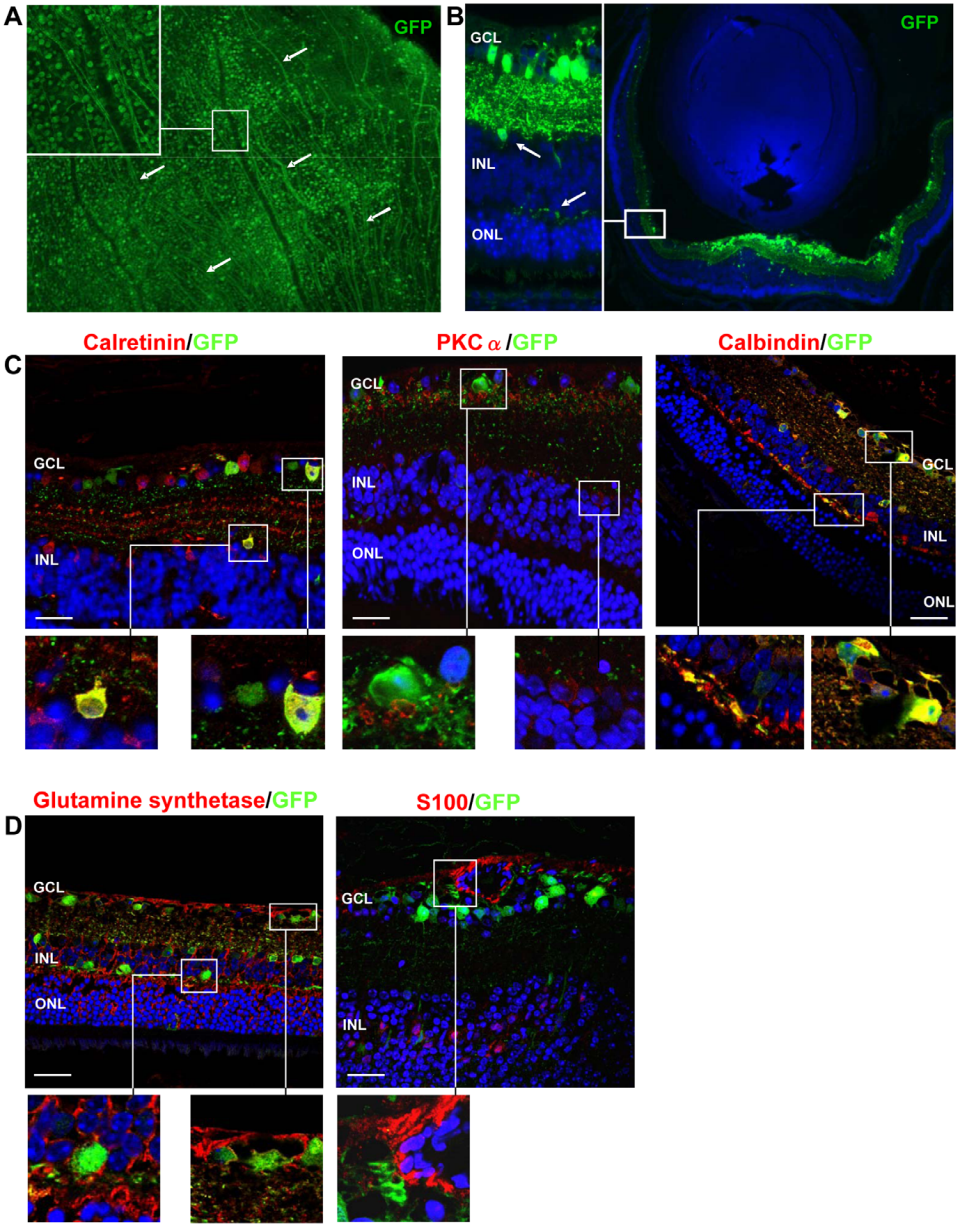
Specific cell types transduced by intravitreal AAV2 in TgIGF-I retinas. (A) Retinal flat-mount immunostained for GFP showing transduction of cells located on the surface of the retina. Arrows indicate GFP signal in axons, demonstrating transduction of ganglion cells. (B) GFP detection in paraffin-embedded eye sections. Besides transduction of cells in the ganglion cell layer (GCL), there was, to a lesser extend, transduction of cells of the Inner Nuclear layer (INL) *(inset, arrows)*. (C) Double immunostaining for GFP and calretinin (amacrine neurons) showed that amacrine cells located both in the surface of the retina as well as in the INL were transduced by AAV2-GFP. Bipolar cells, stained with an antibody against PKC-α, do not show colocalization with GFP nor in the cell bodies localized in the INL *(right inset)* nor in the neuronal projections ending at the GCL *(left inset)*. Immunohistochemistry for calbindin, expressed in horizontal cells of the INL outmost layer *(left inset)* and in a subtype of amacrine cells located in the INL and GCL *(right inset)*, showing that both cell types are transduced by AAV2-GFP. (D) Double immunostaining for GFP and Glutamine synthetase (Müller cells). No expression of GFP was observed in the Müller cells' end feet processes at the retinal surface *(right panel)* or in the cell bodies in the INL *(left panel)*. Astrocytes (S100+), localized in the most superficial retinal layer and around blood vessels, were not transduced by AAV2-GFP. Original magnification: 10× (A), 2× (B). Scale bar: 33 µm (C, *left and central panel*); 74 µm (C, *right panel*); 74 µm (D, *left panel*); 18 µm (D, *right panel*).

The tropism of AAV2 vectors after intravitreal injection has previously been described for wild-type mouse retina [3]. To determine whether the pathological features of TgIGF-I retinas [19], [20] could alter the normal pattern of infection of AAV2 vectors, the expression of the reporter protein GFP was analysed after intravitreal delivery of AAV2-GFP (6.2×10^7^ vg/eye) in which the GFP gene was under the control of an ubiquitous promoter. The majority of the cells on the retinal surface expressed GFP, as shown in flat-mounted retinas (Figure 1A). The protein was also detected in superficial axons, indicating the transduction of ganglion cells whose axonal fibres converge to form the optic nerve (Figure 1A, arrows). GFP was also detected in cells located deeper in the retina (Figure 1B). Detailed immunostaining with specific markers for each neuronal type demonstrated that amacrine (calretinin positive) and horizontal cells (stained with calbindin) were also transduced by AAV2 in IGF-I mice, but neither bipolar cells(specifically detected with a PKCα antibody), nor glial cells (S100 for astrocytes, glutamine synthetase for Müller cells) expressed GFP (Figure 1C, D), probably due to the lack of AAV2-specific receptors on their cell-surfaces. Collectively, the data indicate that intravitreal injection of AAV2 results in efficient transduction of TgIGF-I retinas.

**Figure 2:**
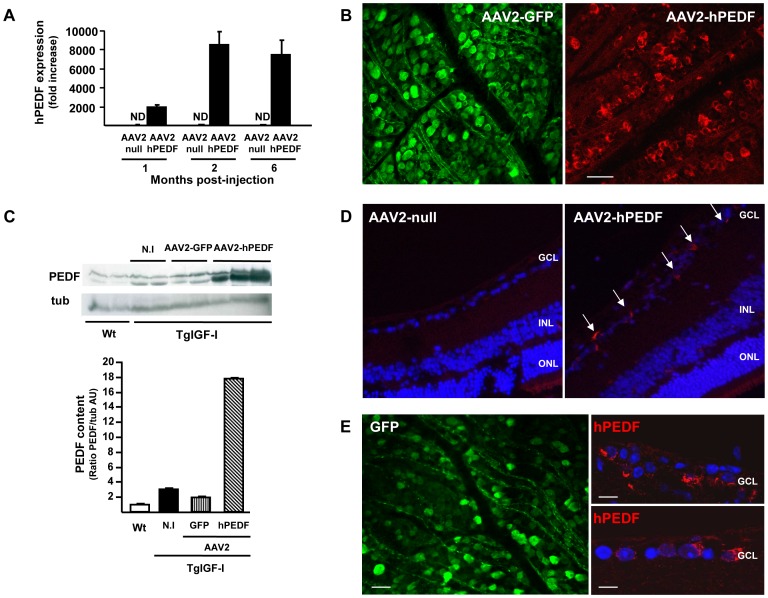
Long-term expression of hPEDF after single AAV2-mediated intravitreal gene delivery. (A) Time-course of hPEDF expression after a single intravitreal delivery of AAV2-hPEDF. Quantitative real time PCR analysis with transgene-specific primers revealed that hPEDF expression reached a plateau 2 months after a single injection of AAV2-hPEDF to 1.5-month-old TgIGF-I mice. hPEDF expression was undetectable in AAV2-null injected contralateral retinas. Values are expressed as the mean ±SEM of 3 animals/group. (B) Immunohistochemical detection of GFP *(left panel)* and PEDF *(right panel)* in retinal flat mounts 6 months after a single intravitreal injection of AAV2-GFP or AVV2-hPEDF demonstrating the expression of the exogenous transgenes. Whereas GFP is an intracellular protein that accumulates inside the cell, allowing the detection of the protein in neuronal dendrites and axons, PEDF is rapidly secreted after production, resulting in a more diffusse staining. Scale bar: 33 µm (C) PEDF Western Blot in retinas of transgenic treated mice. Wild-type, non-injected (N.I.) and AAV2-GFP-treated transgenic mice were used as controls. Values are expressed as the mean ±SEM of 4–6 animals/group. D) Immunodetection of human PEDF in AAV2- null *(left panel)* and AAV2-hPEDF *(right panel)* injected eyes verified that the PEDF overexpressed in ganglion cells was of vector-derived. Original magnification: 20×. (E) *Left panel*, inmunohistochemical detection of GFP (intracellular protein) in a flat-mounted retina 12 months after a single injection of AAV2-GFP to a wild-type mouse. *Right panels*, hPEDF (secreted protein) inmunodetection in paraffin-embedded eye sections 18 months after a single intravitreal injection of AAV2-hPEDF to TgIGF-I. Scale bars: 66 µm (*left panel*), 105 µm (*right upper panel*), 94 µm (*right lower panel*).

It has been shown that AAV vectors allow stable and prolonged gene expression in the retina of rodents and large animal models [22]. Six week-old TgIGF-I mice received a single intravitreal injection of 6.4×10^8^ vg of an AAV2 vector carrying the human PEDF (hPEDF) cDNA. The contralateral eye was treated with a null AAV2 vector, containing a non-coding expression cassette. A steady level of expression of hPEDF was reached 2 months after a single injection of the vector (Figure 2A). Six months after gene transfer, hPEDF was detected by immunofluorescence in flat-mounted retinas, showing a pattern of expression similar to that observed in AAV2-GFP-injected retinas (Figure 2B). Human PEDF was also detected by Western blot in retinal extracts 6 months after injection of the vector (Figure 2C). Retinal PEDF levels presented more than 6-fold increase in comparison with uninjected retinas (Figure 2C, right panel). Noticeably, non-PEDF treated TgIGF-I mice also showed an increase in endogenous retinal PEDF protein, which cross-reacted with by the antibody directed against the human protein in Western blots. This suggests that the upregulation of PEDF may be one of the mechanisms by which non-treated transgenic eyes attempt to counteract the proangiogenic stimulus present. The use, however, of the same antibody to immunolabel PEDF in retinal cross-sections from AAV2-null and AAV2-hPEDF injected eyes, verified that the PEDF overexpressed in ganglion cells was AAV2-hPEDF vector-derived (Figure 2D). Finally, the immunohistochemichal detection of GFP and hPEDF in retinal cells of animals aged 12 and 18 months, respectively, that received a single injection of vector at 1.5 months of age, confirmed lifelong persistence of expression of the gene of interest after AAV-mediated gene delivery to the rodent retina (Figure 2E).

### PEDF treatment inhibits neovascularization in TgIGF-I eyes

**Figure 3:**
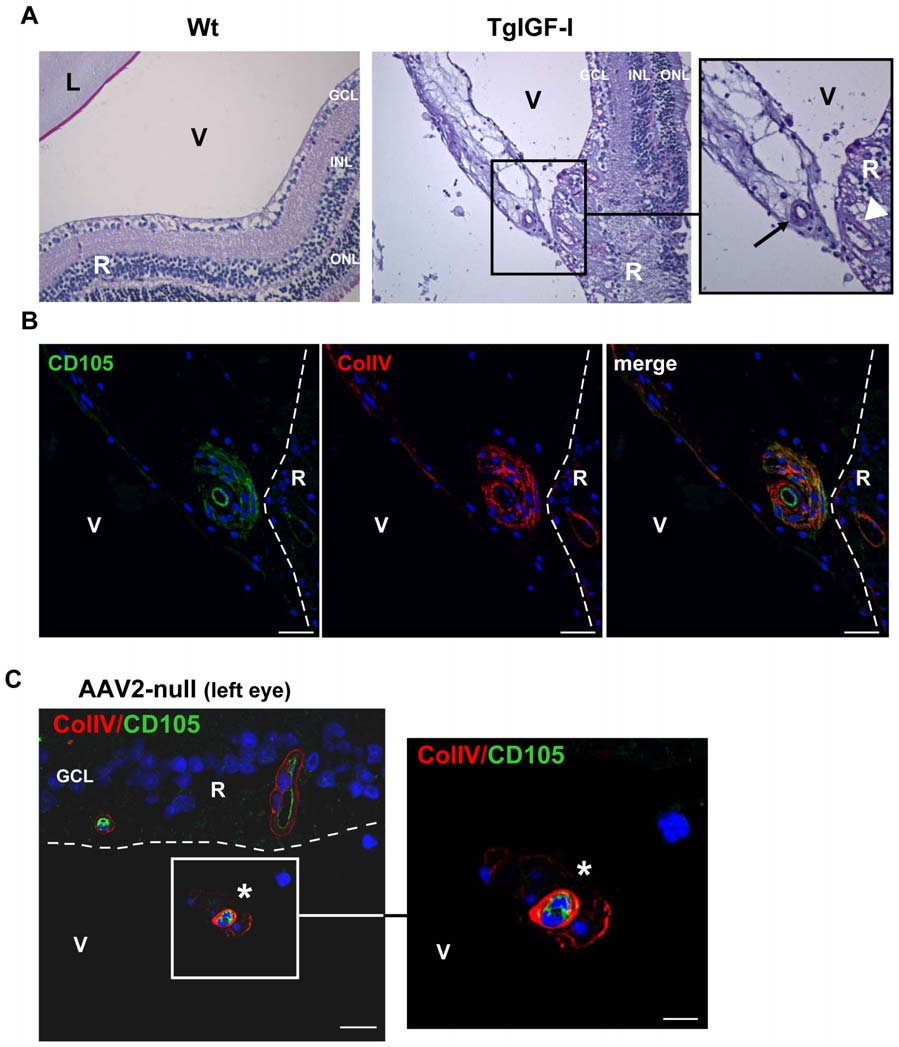
Intravitreal neovessels in non-treated and AAV-null-treated 7.5 month-old TgIGF-I mice. (A) Representative image of PAS-stained sections of transgenic eyes showing intravitreal vessels. Note the presence of fibrotic tissue associated to the vessel attached to the inner limiting membrane of the retina, in an area where the retinal morphology appears altered. Wild-type eyes did not present vascular structures nor fibrosis in the vitreous cavity. Original magnification: 20× (B) ColIV (basal membrane marker) and CD105 (EC marker) inmunostaining in the same intravitreal neovessel depicted in (A) corresponding to a transgenic mouse aged 7.5 months. A clear CD105+ ring-shaped signal, indicating the presence of a lumen, was surrounded by Collagen IV+ staining. Arrows indicate completely formed intravitreal neovessels. Arrowheads indicate a blood vessel within the retina. Scale bar: 25 µm. (C) Representative image of the CD105+/Col-IV+ vascular structures *(asterisks)* present in the vitreous cavity of AAV2null-injected eyes 6 months after delivery of the vectors. No such structures were observed in the contralateral PEDF-treated eyes *(data not shown)*. The dashed line indicates the limit between the retina and the vitreous. Scale bar: 25 µm (*left pannel*), 7.5 µm (*right pannel*). *GCL*, ganglion cell layer; *L*, lenses, *V*, vitreous cavity, *R*, retina.

**Figure 4:**
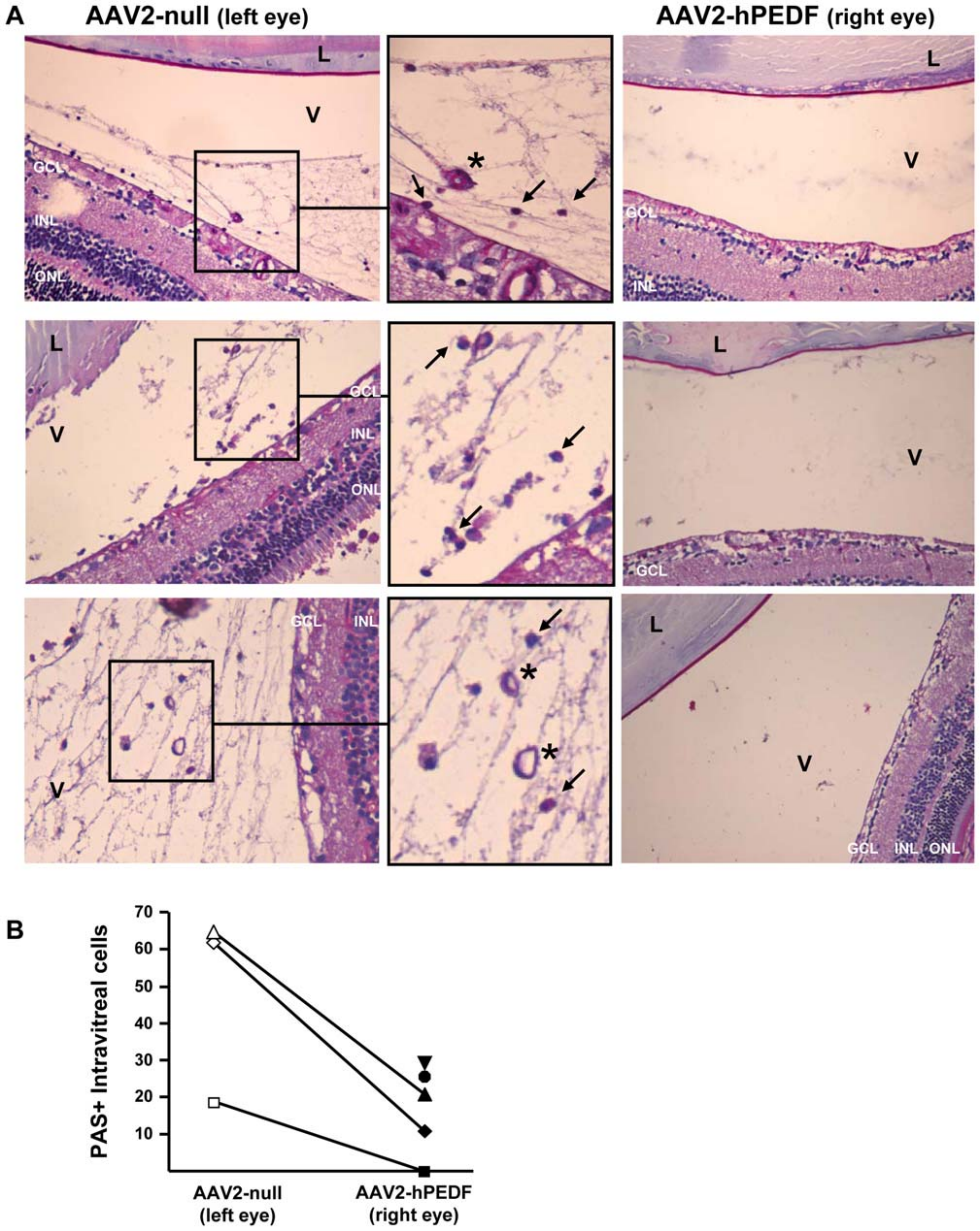
Inhibition of intravitreal neovascularization by AAV2-hPEDF treatment. (A) Representative micrographs of PAS-haematoxylin stained sections of the left (AAV2null-injected) and right (AAV2-hPEDF-injected) eyes from TgIGF-I mice 6 months after delivery of the vectors. Each pair of images corresponds to one animal. Note the presence of completely formed neovessels *(insets, asterisks)* in the vitreous cavity of AAV2-null-injected eyes. Endothelial cells stain strongly for PAS *(inset, arrows)* [21]. Original magnification 20× (B) Endothelial cells on the vitreal side of the internal limiting membrane were counted in six non-consecutive sections per eye. AAV2-hPEDF treated eyes showed a striking reduction in the number of intravitreal PAS positive cells when compared with their untreated contralateral eyes. Two null-injected transgenic eyes could not be analysed due to marked retinal detachment that impeded the counting of PAS+ cells in the vitreous cavity. *GCL*, ganglion cell layer; *INL*, inner nuclear layer; *ONL*, outer nuclear layer; *L*, lenses; *V*, vitreous.

The intraocular accumulation of IGF-I in our transgenic model results in abnormal growth of neovessels in the vitreous cavity [19]. Figure 3A depicts an intravitreal vessel from a non-treated IGF-I transgenic mouse. This completely formed vascular structure is associated with tissue, likely part of a fibrotic inner limiting membrane, attached to a profoundly altered area of the retinal surface. The presence of fully formed vessels in the vitreous cavity of transgenic IGF-I mice was further confirmed by staining with CD105, a marker of endothelial cells [23], and Collagen Type IV, a marker of vascular basement membrane (Figure 3B). Six-months after treatment, AAV-injected eyes were also investigated for the presence of intravitreal vessels. CD105+/Col-IV+ vascular structures were detectable in the vitreous cavity of all transgenic eyes receiving AAV2-null empty vector (Figure 3C), but no vessels were observed in the vitreous cavity of any of the contralateral eyes treated with AAV2-PEDF (data not shown). Similarly, after PAS-haematoxylin staining, no vessels were observed in the vitreous cavity of the right, PEDF-injected eyes (Figure 4A), and the quantification of intravitreal PAS+ cells revealed that hPEDF gene delivery led to a striking reduction in the number of these cells, likely vascular cells [21], with respect to the untreated contralateral eye in all animals analyzed (Figure 4B). Two null-injected transgenic eyes could not be analysed due to marked retinal detachment that impeded the counting of PAS+ cells in the vitreous cavity.

**Figure 5:**
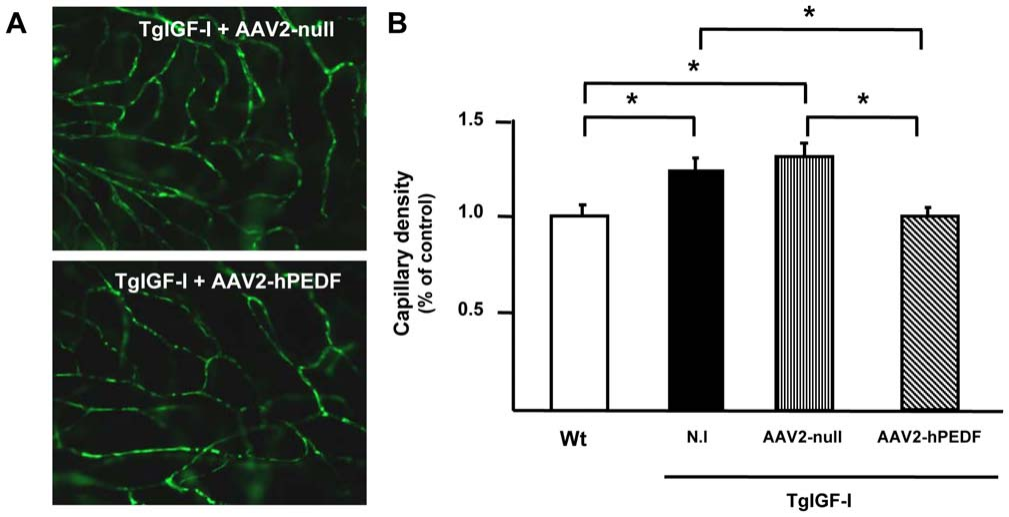
Inhibition of intraretinal neovascularization by AAV2-hPEDF treatment. (A) Representative images used for the quantitative assessment of intrarretinal capillary density. Original magnification: 40×. (B) Twenty high-magnification images of the capillary network that excluded major vessels were taken per retinal angiography with fluorescein-conjugated dextran, and the area corresponding to the capillary area was calculated using software tools as percentage of green area over total retinal area. Digital image quantification showed a significant increase in the retinal capillary density in transgenic animals compared with Wt. Six months after a single treatment with AAV2-hPEDF transgenic retinas showed a capillary density similar to that of Wt healthy retinas. N.I., non-injected. Values are expressed as the mean ±SEM of 7 animals/group. *P<0.05.

To quantify the inhibitory effects of hPEDF on the intraretinal vasculature, we measured the retinal capillary density at 7.5 months of age by digital image analysis of fluorescein angiographies at high magnification (Figure 5A). TgIGF-I mice showed an increment of 20% in capillary density in comparison with age-matched wild-type (Wt) animals (Figure 5B). Six months after treatment, transgenic retinas treated with hPEDF presented a capillary density similar to that of healthy retinas. This suggested that vector-mediated PEDF overexpression succeeded in counteracting the ongoing neovascularization process present in transgenic retinas without affecting stable pre-existing vessels.

### Effects of PEDF overexpression on retinal detatchment

**Figure 6:**
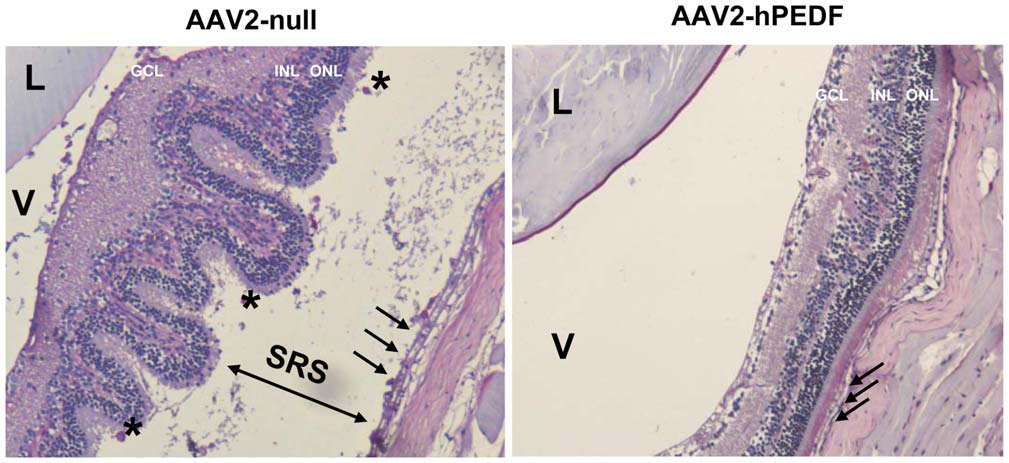
PEDF gene delivery prevents retinal detachment in TgIGF-I. Areas of retinal detachment were identified histologically by the presence of retinal folds. In most of the cases, infiltrating cells were observed in the subretinal space created around the detachment *(left panel, asterisks)*. Arrows indicate the monolayer of cells than constitute the Retinal Pigment Epithelium (RPE). Representative micrographs of the AAV-null and AAV2-hPEDF injected eyes of the same animal are shown. The incidence of retinal detachment was reduced from 83% to 16.5% after PEDF treatment (*n* = 6). *GCL*, ganglion cell layer; *INL*, inner nuclear layer; *ONL*, outer nuclear layer; *L*, lenses; *V*, vitreous; *SRS*, Subretinal space. Original magnification: 10×.

Retinal detachment can occur in advanced diabetic retinopathy as a consequence of intravitreal neovascularization that tractionally detaches the neuroretina from the supporting pigment epithelium leading to visual loss [24]. In agreement with the inhibitory effect of hPEDF on preretinal neovascularization, six months after vector injection the incidence of retinal detachment was 83% for the AAV2-null-treated eyes while it was only of 16.5% for the AAV2-hPEDF-treated eye (Figure 6). These results clearly indicate that hPEDF treatment, either directly or through the inhibition of intravitreal neovascularization is very efficacious in preventing retinal detachment, a sight-threatening complication.

### PEDF overexpression leads to decreased VEGF levels in a hypoxia-independent manner

**Figure 7:**
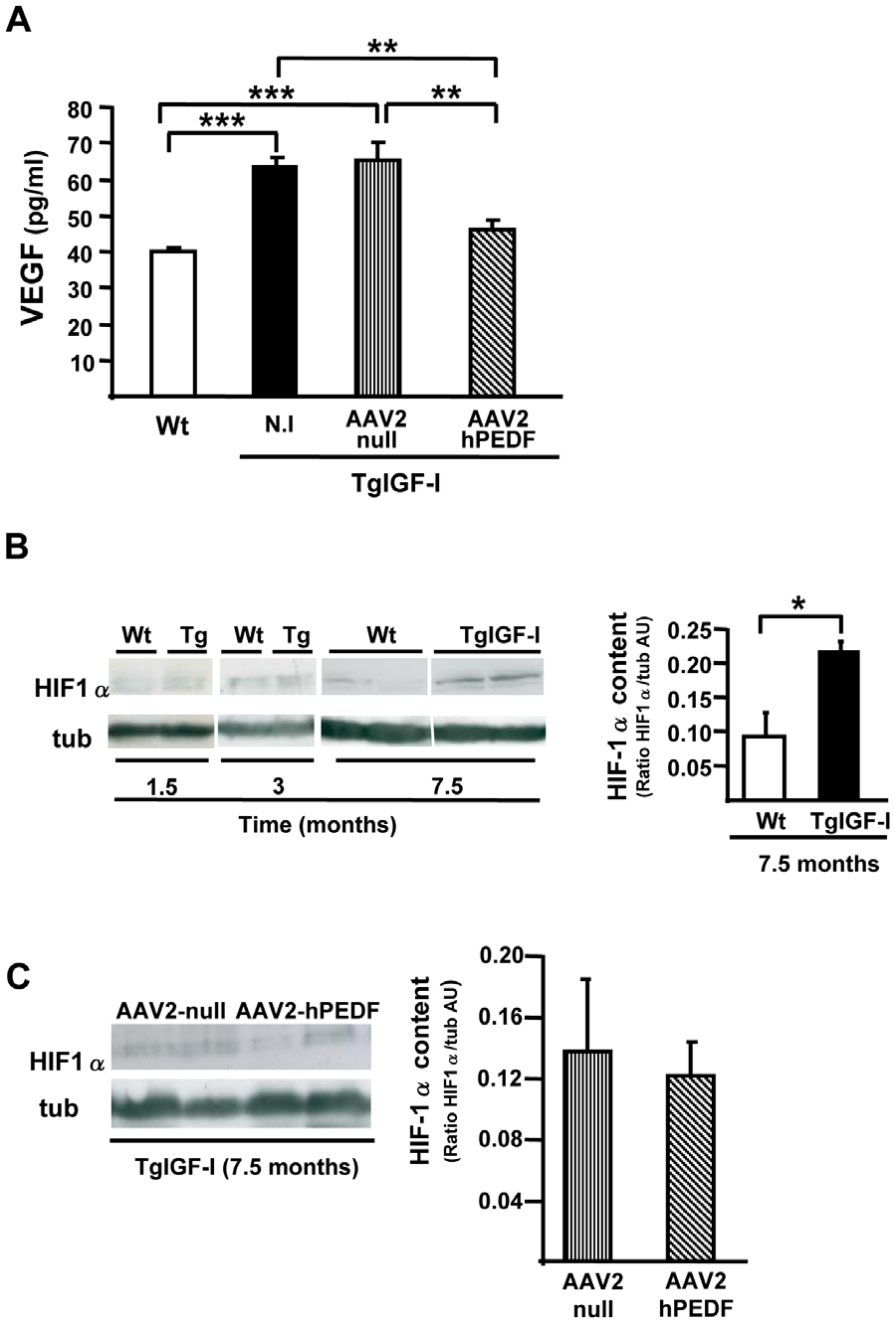
Effects of AAV2-hPEDF treatment on VEGF levels in a HIF-1α independent manner. (A) Detection by ELISA of increased intraocular VEGF levels in both TgIGF-I and AAV2-null injected TgIGF-I mice. Levels normalized 6 months after AAV2-hPEDF treatment. (B) Time-course of retinal HIF-1α expression in TgIGF-I mice. HIF-1α protein was increased in retinas of 7.5 months-old TgIGF-I, remaining unaltered in younger animals. (C) AAV2-PEDF treatment did not affect HIF-1α expression 6 months after injection of the vector. N.I., non-injected. Values are expressed as the mean ±SEM of 4–10 animals/group. ***P<0.001, **P<0.01, *P<0.05.

VEGF is increased in the vitreous of patients with proliferative retinopathy and also in animal models of retinal neovascularization [2], [25], including TgIGF-I mice [19], [20]. VEGF concentration was measured by ELISA in aqueous humour of WT and non-injected TgIGF-I, AAV2-null and AAV2-hPEDF treated TgIGF-I mice at 7.5 months of age. Samples obtained from non-injected and AAV2-null injected TgIGF-I showed a 50% of increment in VEGF levels with respect to Wt (Figure 7A). This increased VEGF in transgenic mice was normalized 6 months after a single treatment with AAV2-hPEDF (Figure 7A), which was consistent with the inhibition of neovascularization observed (Figures 4A, B, 5).

Hypoxia-inducible factor 1α (HIF-1α) is the main regulator of the hypoxic response and promotes neovascularization through upregulation of VEGF [26]. When HIF-1α was analyzed in retinas of TgIGF-I mice at different ages, an aproximately 2-fold increase over Wt levels was observed in 7.5 month-old mice, which was not evident in younger animals (Figure 7B). However, HIF-1α levels remained unchanged six months after AAV2-hPEDF administration in TgIGF-I mice (Figure 7C), suggesting that PEDF inhibitory actions on vascular growth were not mediated by a reduction in HIF-1α.

### PEDF treatment reduces angiogenesis-related factors in TgIGF-I eyes

**Figure 8:**
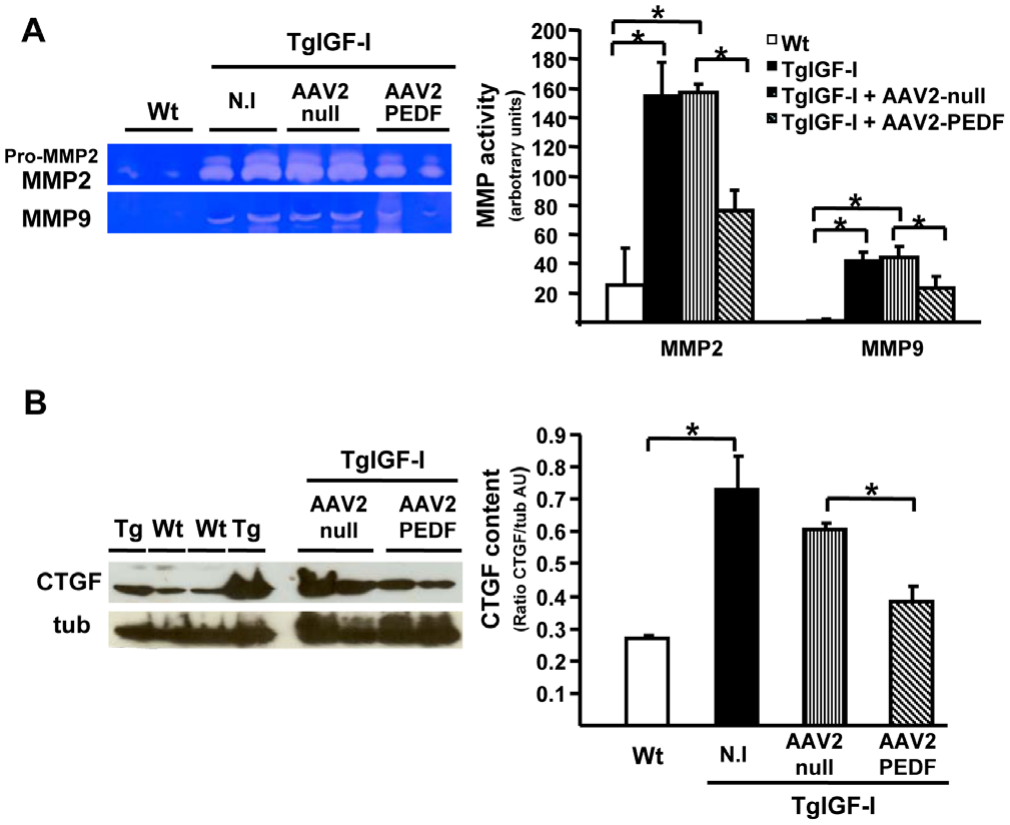
PEDF overexpression downregulates the activity of VEGF- regulated factors. (A) Decreased MMP2 and 9 activity in aqueous humour from TgIGF-I mice after AAV2-hPEDF treatment. (B) Transgenic mice showed increased CTGF accumulation in comparison with WT. CTGF levels were reduced after 6 months of AAV2-hPEDF treatment, but not after AAV2-null injection. N.I., non-injected. Values are expressed as the mean ±SEM of 4–10 animals/group. *P<0.05.

Matrix metalloproteinases (MMPs) are collagen degrading enzymes essential for the remodelling of the vascular basement membrane during neovessel formation. Their expression has been described to be regulated by several factors including VEGF [27]. Consistent with the angiogenic process, TgIGF-I mice had increased intraocular activity of both MMP2 and MMP9 at 7.5 months of age (Figure 8A). Six months after vector injection, a reduction in the activity of these gelatinases was observed in aqueous humour samples from PEDF-treated mice but not from AAV2-null injected mice (Figure 8A). This reduction in metalloproteinases activity probably reflected the normalization of ocular VEGF observed in PEDF-treated animals.

In proliferative retinopathy, neovascularization may progress to a fibrotic phase that has been associated with elevated vitreous levels of Connective Tissue Growth Factor (CTGF) [28]. VEGF directly upregulates CTGF expression in retinal vascular cells [29]. When CTGF protein levels were analyzed by Western blot, TgIGF-I retinas showed an approximately 7-fold increase in this growth factor when compared to Wt (Figure 8B). However, after 6 months of AAV2-hPEDF treatment CTGF levels were normalized (Figure 8B). Thus, PEDF overexpression could be an effective way of preventing neovascularization-related fibrosis.

## Discussion

Effective treatment for sight-threatening retinal neovascularization associated with diseases such as diabetic retinopathy represents a significant unmet medical need. Given the long-term nature of these processes, the assessment of new therapies requires their testing in adequate animal models that follow the pattern of disease progression over time that the human disease does. The present study shows that retinal PEDF gene transfer mediated by AAV vectors results in inhibition of neovascularization in TgIGF-I mice up to 6 months after a single injection.

AAV vectors have extensively been used for retinal transduction in several animal models [3], [4] and in human subjects [5]–[7]. Provided an ubiquitous promoter is used to drive the expression of an exogenous gene, the type of cell which will be transduced by a viral vector (tropism) is specific to the vector serotype and depends on the presence of receptors on the cell surface that mediate viral attachment and entry [30], [31]. Several serotypes of AAVs have been isolated, which show different tissue and cell tropisms allowing for purpose-based selection of vectors [32]. Our choice of AAV2 was based on the ability of this vector to efficiently transduce the cells located on the retinal surface [3], so that these cells would secrete the therapeutic protein in close proximity to the retinal vasculature. However, recent work published by Chen and colleagues confirmed the role of the physiological conditions of a tissue in creating specific molecular signatures that can influence AAV vector tropism [33]. Thus, we studied AAV2 vector tropism in our IGF-I transgenic model. As described for other murine models [3], after intravitreal delivery of an AAV2-GFP, the majority of the cells on the ganglion cell layer expressed the protein, indicating that the intravitreal efficiency of AAV2 vectors was not altered in young TgIGF-I mice. Despite their exposure to the viral solution, glial cells were not transduced by AAV2, as previously reported for healthy rodent retinas [3]. AAV2-hPEDF transduction efficiency was similar to that of AAV2-GFP and persisted throughout the animal's life. Protein expression following intravitreal injection of AAV vectors has been reported to last up to 6 years in large animal models [4]. This long-lasting transgene expression may result from the low immunogenicity of AAV vectors together with the immune privilege present in the eye, which attenuates immune responses that could limit gene transfer efficacy [34]. Hence, one single injection of AAV2-hPEDF allows stable expression of the therapeutic protein, thus avoiding the potentially deleterious effects of periodic intraocular injections of therapeutic agents required for the treatment of chronic pathologies like diabetic retinopathy.

AAV-PEDF gene therapy has proved successful in other animal models of ocular neovascularization, such as laser-induced choroidal neovascularization [35] and oxygen-induced retinopathy [36]. However, these models have an acute and early regressive phenotype that does not make them ideal for long-term evaluations, required when the target pathology has slow development and chronic features, as diabetic retinopathy or macular oedema. In contrast, in the model used in the current study, the TgIGF-I model, retinal neovascularization develops over time as animals age, and is a result of intraocular IGF-I and VEGF lifelong accumulation [19], [20]. Although the levels of intraocular IGF-I in our model are high compared to values reported in human proliferative DR (∼40% vs ∼2–5% of circulating levels, respectively [37], [38]), vascular alterations develop slowly as animals age, despite the presence of the growth factor from birth. Retinal capillary density is significantly increased in our animal model at 7.5 months of age when compared with age-matched Wt animals. Transgenic animals receiving AAV2-hPEDF treatment at 1.5 months of age showed normalization of the capillary density 6 months after gene transfer. Moreover, PEDF treatment led to a striking decrease in the number of vascular cells detected in the vitreous cavity of these mice, which was associated with a marked reduction in the incidence of retinal detachment. Altogether these data suggest that PEDF overexpression can counteract the ongoing intraretinal and intravitreal neovascularization that develops with age in TgIGF-I mice. Although PEDF expression by ganglion cells in the foveal region is proposed to define the foveal avascular area [39], the fact that the capillary density in PEDF overexpressing retinas was similar to that of healthy Wt retinas, and not less, agrees with published literature supporting the notion that PEDF effects are restricted to newly formed vessels and do not affect quiescent vessels in the adult retina [13], [35].

The molecular mechanisms through which PEDF exerts its antiangiogenic actions still remain elusive. PEDF induces apoptosis of endothelial cells and inhibits their migration [40]. Recent evidence suggests that PEDF action may be based on the disruption of VEGF signalling, impairing its binding to VEGF-R2 [41] or degrading VEGF-R1 [42]. TgIGF-I mice have increased intraocular levels of VEGF [19], [20], which were normalized after hPEDF overexpression. This effect on VEGF protein was independent of HIF-1α, a regulator of VEGF expression under hypoxia [43], as indicated by the fact that the level of this protein remained unaltered after hPEDF treatment. Altogether, these observations suggest that PEDF may be acting by either regulating VEGF expression or VEGF stability. In addition, the ability of PEDF to regulate VEGF levels and inhibit angiogenesis despite the presence of increased HIF-1α suggests that overexpression of PEDF could inhibit neovascularization in hypoxic environments.

Noteworthy, we found that PEDF treatment did not lead to complete inhibition of VEGF; after months of continuous PEDF expression VEGF levels in AAV-hPEDF-treated mice were similar to those of Wt. Although short-term clinical studies suggest that anti-VEGF therapies may have some beneficial effects in the treatment of proliferative diabetic retinopathy (reviewed in [44]) results are not conclusive. Basal levels of VEGF may be crucial to ensure the survival of retinal neurons and glial cells, as evidenced by studies with intensive anti-VEGF therapies that underscored the role of VEGF as a neuroprotective molecule [45]. In this regard, we believe that PEDF might be a safer candidate, and our results indicate that PEDF overexpression normalizes VEGF levels to those found in healthy retinas. Thus, our study in an animal model with slow-progression retinopathy reveals PEDF-gene transfer as a potentially useful therapy for ischemic retinopathies, which can target neovascularization without compromising protective VEGF levels. This may be important for diseases, such as diabetic retinopathy, which require lifelong treatment, and in which retinal neuronal function is also compromised [46]. Furthermore, PEDF has been reported to have anti-inflammatory and neuroprotective properties, which would be of added value to its antiangiogenic potential [14]–[17].

VEGF is the main activator of the proangiogenic process and regulates the expression of other factors implicated in neovessel formation. Amongst them, MMP2 and MMP9 are responsible for the ECM remodelling needed for retinal neovascularization [47]. Their activity is increased in TgIGF-I eyes and decreased after hPEDF treatment, suggesting that VEGF signalling is also reduced after PEDF overexpression. Furthermore, CTGF, another downstream effector of VEGF whose increase in retinopathy underlies fibrotic processes associated with retinal angiogenesis [28], was also normalized in AAV-hPEDF treated retinas. This anti-fibrogenic activity has been previously reported after PEDF gene transfer in kidney of diabetic rats [48] but never before for the eye. Together, these results show that PEDF overexpression is able to counteract the neovascularization process present in TgIGF-I retinas by modulating the expression of angiogenesis-related factors.

In summary, in this study we showed that PEDF was able to counteract angiogenesis in an adult and chronic model of retinal neovascularization after a single injection of AAV2-hPEDF, instead of repeated administrations of the therapeutic agent. In addition, AAV-mediated gene transfer of hPEDF to the eye results in normalization of intraocular levels of VEGF and other pro-angiogenic molecules. These findings suggest that AAV-hPEDF treatment may represent an effective therapy for diabetic retinopathy and other ocular diseases characterized by neovascularization.

## Materials and Methods

### Animals

CD1 heterozygous mice overexpressing IGF-I in the retina were used.

### Ethics statement

Animal care and experimental procedures were approved by the Ethics Committee in Animal and Human Experimentation of Universitat Autònoma de Barcelona (UAB).

### Vector production

Vectors were generated by helper virus-free transfection of HEK293 cells (Q-Biogene, Montreal, Canada) with three plasmids: (1) a vector plasmid carrying the expression cassette flanked by the viral ITRs corresponding to AAV2; (2) a packaging plasmid carrying the AAV2 rep and cap genes; and (3) a helper plasmid carrying the adenovirus helper functions. The expression cassette contained the ubiquitous CAG promoter (hybrid of CMV early enhancer/chicken ß actin promoter), GFP or human PEDF cDNA (provided by Dr. Susan Crawford, Northwestern University, Evanston, IL, USA) and WPRE element. Three days after transfection, cells were collected and lysated. Viral particles were purified by iodixanol gradient.

### Vector administration

Under a dissecting microscope, 2 µl of viral solution were delivered to the vitreous cavity of deeply anesthetized mice (100 mg/kg ketamine/10 mg/kg xylazine) using a 10-mm 33-gauge needle mounted on a 5 µl Hamilton syringe (Hamilton Bonaduz AG, Switzerland). The needle was advanced through the sclera near to the corneoscleral limbus. Pupils were previously dilated with Tropicamide (Alcon Cusí, El Masnou, Spain) and antibiotic and anti-inflammatory drops (Tobradex, Alcon Cusí, El Masnou , Spain) were administered after the injection. Animals showing signs of trauma or ocular bleeding after the injection were excluded from the study.

### RNA extraction and quantitative real-time PCR

Retinas were homogenized in 200 µl of Tripure and retinal RNA was purified using RNeasy Mini Kit (QIAGEN Sciences, Germantown, MD, USA). cDNA was synthesized with Transcriptor First Strand cDNA Synthesis Kit (Roche, Basel, Switzerland). Quantitative real-time PCR was performed using LightCycler® 480 SYBR Green I Master (Roche, Basel, Switzerland) with hPEDF-specific primers: Fw-TACCGGGTGCGATCCAGCA and Rv-TGGGCTGCTGATCAAGTCA (Invitrogen, Paisley, UK).

### Histological analysis

Formalin-fixed paraffin-embedded eye sections were incubated with anti-GFP (ab6673, Abcam, Cambridge, UK; diluted 1/300), anti-calretinin (7699/4, Zymed Laboratories, Invitrogen, Paisley, UK; diluted 1/100), anti-PKCα (P 5704, Sigma-Aldrich, St. Louis, MO, USA; diluted 1/500), anti-calbindin (C 7354, Sigma-Aldrich, St. Louis, MO, USA; diluted 1/5000), anti-glutamine synthetase (G 2781, Sigma-Aldrich, St. Louis, MO, USA; diluted 1/500), anti-S100 (Z 0311, DAKO, Glostrup, Denmark; diluted 1/500), anti-CollagenIV (AB756P, Chemicon, Millipore, Billerica, MA, USA, diluted 1/100) or anti-CD105 (BAF1320, R&D Systems, Minneapolis, MN, USA; diluted 1/10). Whole-mount formalin-fixed retinas were incubated with anti-GFP (diluted 1/300) or anti-PEDF (AF1177, R&D Systems, Minneapolis, MN, USA; diluted 1/50). Periodic acid-Schiff (PAS) and hematoxylin staining was performed in paraffin-embedded eye sections. Images were obtained with a laser-scanning confocal microscope (TCs SP2; Leica Microsystems GmbH, Wetzlar, Germany) or a Nikon Eclipse 90i microscope (Nikon Instruments Inc., Melville, NY, USA).

### Protein analysis

For Western blot, retinas were homogenized in lysis buffer and 100 µg of retinal extract were separated by 12% SDS-PAGE. Immunoblot was performed with anti-PEDF (AF1177, R&D Systems, Minneapolis, MN, USA; working dilution 1/100), anti-CTGF (ab5097, Abcam, Cambridge, UK; working dilution 1/50), or anti-Tubulin (ab4074, Abcam, Cambridge, UK; working dilution 1/500). Detection was performed using Immobilon Western reagent (Millipore, Billerica, MA, USA). The pixel intensity of the bands obtained was determined with GeneSnap software for Gene Genius Bio Imaging System (Syngene, Synoptics Ltd, Cambridge, UK) and the ratio protein content/tubulin content was calculated for each sample to allow loading-independent comparison. For VEGF detection in aqueous humor 10 µl of sample were analysed with the Mouse VEGF ELISA kit (QIA52, Calbiochem, EMD Biosciences, Inc., Gibbstown, NJ, USA).

### Assessment of Neovascularization

For retinal angiograms, animals received a tail vein injection of 50 mg/ml FITC-conjugated Dextran (Sigma-Aldrich, St. Louis, MO, USA ). Ten minutes later, animals were sacrificed and retinas were dissected, flat-mounted and fixed. Twenty images per retina (40×) were captured (Nikon Eclipse 90i, Nikon Instruments Inc., Melville, NY, USA) and capillary area was determined using NIS Elements Advanced Research 2.20 software (Nikon Instruments Inc., Melville, NY, USA). Pre-retinal vessels were analyzed in PAS-stained eye sections [21]. The number of PAS+ intravitreal endothelial cells, on the vitreal side of the internal limiting membrane, was counted in 6 non-consecutive sections/eye.

### Zymography

3 µl of aqueous humor were directly loaded into 12% polyacrylamide running gels containing 0.1 mg/ml of porcine gelatin (BioRad, Hercules, CA, USA). Gels were removed from glass plates, rinsed and incubated in 1 M Tris-HCl pH 7.5, 0.5 M CaCl_2_ for 18 h at 37°C. After incubation, gels were stained in 0.1% Coomassie Blue, 10% acetic acid, 10% isopropanol for 2 h and then destained in 10% acetic acid, 10% isopropanol until the bands were clearly visible.

### Statistical analysis

Values are expressed as the mean ± SEM. Differences between groups were compared by unpaired Student *t* test. Differences were considered statistically significant at *P* values less than 0.05.
